# 1,2-Dimeth­oxy-3-[(*E*)-2-nitro­ethen­yl]benzene

**DOI:** 10.1107/S1600536810030539

**Published:** 2010-08-11

**Authors:** Yuehong Ren, Ruitao Zhu

**Affiliations:** a Department of Chemistry, Taiyuan Normal University, Taiyuan 030031, People’s Republic of China

## Abstract

The title compound, C_10_H_11_NO_4_, was synthesized *via* condensation of 2,3-dimeth­oxy­benzaldehyde with nitro­methane using microwave irradiation without solvent. The H atoms of the –CH=CH– group are in a *trans* configuration. The dihedral angle between the mean planes of the benzene ring and the nitro­alkenyl group is 23.90 (6)°.

## Related literature

For the use of nitro­alkenes in organic synthesis, see: Ranu & Banerjee (2005 ▶); Ballini *et al.* (2005 ▶). For a related structure, see: Pedireddi *et al.* (1992 ▶). For the synthetic procedure, see: Wang & Wang (2002 ▶).
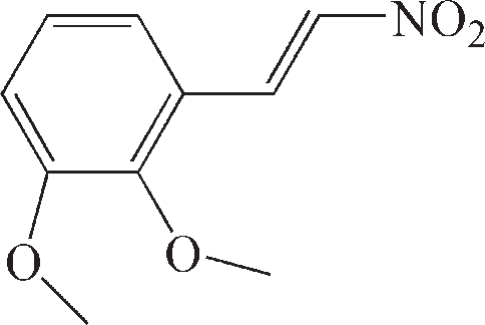


## Experimental

### 

#### Crystal data

C_10_H_11_NO_4_
*M* *_r_* = 209.20
Monoclinic, 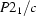

*a* = 5.3558 (7) Å
*b* = 13.5897 (11) Å
*c* = 14.2646 (12) Å
β = 97.038 (1)°
*V* = 1030.41 (18) Å^3^
*Z* = 4
Mo *K*α radiation
μ = 0.11 mm^−1^
*T* = 296 K
0.38 × 0.35 × 0.34 mm

#### Data collection

Bruker APEXII CCD diffractometer
Absorption correction: multi-scan (*SADABS*; Sheldrick, 1996 ▶) *T*_min_ = 0.961, *T*_max_ = 0.965
4852 measured reflections
1798 independent reflections
1351 reflections with *I* > 2σ(*I*)
*R*_int_ = 0.035

#### Refinement

*R*[*F* ^2^ > 2σ(*F* ^2^)] = 0.058
*wR*(*F* ^2^) = 0.226
*S* = 1.14
1798 reflections
139 parameters
H-atom parameters constrained
Δρ_max_ = 0.41 e Å^−3^
Δρ_min_ = −0.41 e Å^−3^

### 

Data collection: *APEX2* (Bruker, 2007 ▶); cell refinement: *SAINT* (Bruker, 2007 ▶); data reduction: *SAINT*; program(s) used to solve structure: *SHELXS97* (Sheldrick, 2008 ▶); program(s) used to refine structure: *SHELXL97* (Sheldrick, 2008 ▶); molecular graphics: *SHELXTL* (Sheldrick, 2008 ▶); software used to prepare material for publication: *SHELXTL*.

## Supplementary Material

Crystal structure: contains datablocks I, global. DOI: 10.1107/S1600536810030539/lh5097sup1.cif

Structure factors: contains datablocks I. DOI: 10.1107/S1600536810030539/lh5097Isup2.hkl

Additional supplementary materials:  crystallographic information; 3D view; checkCIF report

## Figures and Tables

**Fig. 1.**
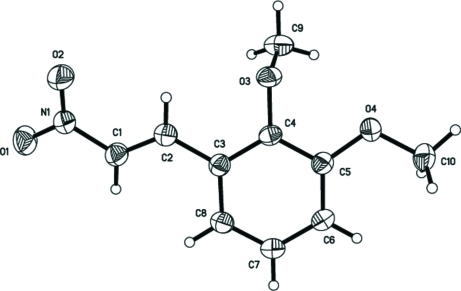
A view of the molecular structure of the title compound, displacement ellipsoids are drawn at the 30% probability level.

## supplementary crystallographic information

### Comment

Nitroalkenes are good substrates for Michael addition reactions because of the stronger electron withdrawing property of the nitro group (Ranu *et al.*, 2005). In addition, the nitro group can provide a good nitrogen source for the synthesis of many useful organic molecules (Ballini *et al.*, 2005). Our group has focused on new organic transformations obtained by nitroalkenes as substrates. In this paper, we report the structure of the title compound. The crystal structure of the title compound is shown in Fig. 1. The H atoms of the -CH═CH- group are in a trans configuration. The dihedral angle between the mean planes of the benzene ring and the nitroalkenyl group is 23.90 (6)°. The bond lengths and angles in the tilte compound can be compared to those in (E)-β-nitrostyrene (Pedireddi *et al.*, 1992).

### Experimental

The title compound was prepared according to a method reported in the literature (Wang *et al.* (2002): A mixture of 0.83 g (5 mmol) 2,3-dimethoxy-benzaldehyde, 1.53 g (25 mmol) nitromethane and 0.35 g potassium carbonate was finely ground by agate mortar and pestle and was mixed with 5 g aluminium oxide (150mesh). The mixture was then put in a 25 ml beaker and introduced into a microwave oven. Microwave irradiation was carried out for 5 min. The mixture was cooled to ambient temperature, then water and nitromethane were removed by reduced pressure. The residue was purified by silica gel chromatography (pertroleum ether/ethyl acetate/dichloromethane. 1:1:0.3) to give the product (yield 75%). Crystals suitable for X-ray analysis were obtained after one week by slow evaporation from an ethyl alcohol solution of the title compound.

### Refinement

H atoms were placed in idealized positions and allowed to ride on their respective parent atoms, with C—H = 0.93-0.96 Å and with *U*_iso_(H) = 1.2*U*_eq_(C) or 1.2*U*_eq_(C_methyl_).

### Crystal data

**Table:**

C_10_H_11_NO_4_	*F*(000) = 440
*M**_r_* = 209.20	*D*_x_ = 1.349 Mg m^−^^3^
Monoclinic, *P*2_1_/*c*	Mo *K*α radiation, λ = 0.71073 Å
Hall symbol: -P 2ybc	Cell parameters from 1678 reflections
*a* = 5.3558 (7) Å	θ = 3.0–25.5°
*b* = 13.5897 (11) Å	µ = 0.11 mm^−^^1^
*c* = 14.2646 (12) Å	*T* = 296 K
β = 97.038 (1)°	Flake, colorless
*V* = 1030.41 (18) Å^3^	0.38 × 0.35 × 0.34 mm
*Z* = 4	

### Data collection

**Table:**

Bruker APEXII CCD diffractometer	1798 independent reflections
Radiation source: fine-focus sealed tube	1351 reflections with *I* > 2σ(*I*)
graphite	*R*_int_ = 0.035
φ and ω scans	θ_max_ = 25.0°, θ_min_ = 2.1°
Absorption correction: multi-scan (*SADABS*; Sheldrick, 1996)	*h* = −6→6
*T*_min_ = 0.961, *T*_max_ = 0.965	*k* = −16→15
4852 measured reflections	*l* = −15→16

### Refinement

**Table:**

Refinement on *F*^2^	Secondary atom site location: difference Fourier map
Least-squares matrix: full	Hydrogen site location: inferred from neighbouring sites
*R*[*F*^2^ > 2σ(*F*^2^)] = 0.058	H-atom parameters constrained
*wR*(*F*^2^) = 0.226	*w* = 1/[σ^2^(*F*_o_^2^) + (0.1507*P*)^2^] where *P* = (*F*_o_^2^ + 2*F*_c_^2^)/3
*S* = 1.14	(Δ/σ)_max_ < 0.001
1798 reflections	Δρ_max_ = 0.41 e Å^−^^3^
139 parameters	Δρ_min_ = −0.41 e Å^−^^3^
0 restraints	Extinction correction: *SHELXL97* (Sheldrick, 2008), Fc^*^=kFc[1+0.001xFc^2^λ^3^/sin(2θ)]^-1/4^
Primary atom site location: structure-invariant direct methods	Extinction coefficient: 0.17 (3)

### Special details

**Table:**

Geometry. All e.s.d.'s (except the e.s.d. in the dihedral angle between two l.s. planes) are estimated using the full covariance matrix. The cell e.s.d.'s are taken into account individually in the estimation of e.s.d.'s in distances, angles and torsion angles; correlations between e.s.d.'s in cell parameters are only used when they are defined by crystal symmetry. An approximate (isotropic) treatment of cell e.s.d.'s is used for estimating e.s.d.'s involving l.s. planes.
Refinement. Refinement of *F*^2^ against ALL reflections. The weighted *R*-factor *wR* and goodness of fit *S* are based on *F*^2^, conventional *R*-factors *R* are based on *F*, with *F* set to zero for negative *F*^2^. The threshold expression of *F*^2^ > σ(*F*^2^) is used only for calculating *R*-factors(gt) *etc*. and is not relevant to the choice of reflections for refinement. *R*-factors based on *F*^2^ are statistically about twice as large as those based on *F*, and *R*- factors based on ALL data will be even larger.

### Fractional atomic coordinates and isotropic or equivalent isotropic displacement parameters (Å^2^)

**Table:**

	*x*	*y*	*z*	*U*_iso_*/*U*_eq_	
N1	0.8816 (4)	0.34090 (16)	0.43863 (15)	0.0521 (7)	
O1	1.0954 (4)	0.35541 (16)	0.47743 (16)	0.0740 (7)	
O2	0.7912 (4)	0.25907 (15)	0.42869 (17)	0.0818 (8)	
O3	0.1183 (3)	0.38811 (12)	0.21248 (12)	0.0532 (6)	
O4	−0.2003 (3)	0.53204 (13)	0.14689 (13)	0.0581 (6)	
C1	0.7376 (5)	0.4265 (2)	0.40583 (19)	0.0544 (7)	
H1A	0.7990	0.4887	0.4234	0.065*	
C2	0.5216 (5)	0.4187 (2)	0.35173 (16)	0.0503 (7)	
H2	0.4684	0.3556	0.3335	0.060*	
C3	0.3580 (4)	0.50020 (18)	0.31776 (16)	0.0454 (7)	
C4	0.1580 (4)	0.48261 (15)	0.24744 (16)	0.0439 (7)	
C5	−0.0092 (4)	0.55801 (18)	0.21569 (17)	0.0470 (7)	
C6	0.0260 (5)	0.65146 (18)	0.2532 (2)	0.0532 (7)	
H6	−0.0844	0.7019	0.2324	0.064*	
C7	0.2273 (5)	0.6697 (2)	0.3224 (2)	0.0583 (8)	
H7	0.2515	0.7328	0.3470	0.070*	
C8	0.3908 (5)	0.5959 (2)	0.35469 (18)	0.0546 (7)	
H8	0.5237	0.6092	0.4012	0.066*	
C9	0.2010 (7)	0.3753 (2)	0.1216 (2)	0.0716 (9)	
H9A	0.1121	0.4202	0.0774	0.107*	
H9B	0.1682	0.3090	0.1005	0.107*	
H9C	0.3783	0.3882	0.1259	0.107*	
C10	−0.3741 (5)	0.6077 (2)	0.11368 (19)	0.0605 (8)	
H10A	−0.4538	0.6327	0.1655	0.091*	
H10B	−0.4994	0.5811	0.0666	0.091*	
H10C	−0.2858	0.6600	0.0867	0.091*	

### Atomic displacement parameters (Å^2^)

**Table:**

	*U*^11^	*U*^22^	*U*^33^	*U*^12^	*U*^13^	*U*^23^
N1	0.0511 (13)	0.0521 (13)	0.0517 (13)	0.0009 (9)	0.0004 (10)	0.0000 (9)
O1	0.0514 (13)	0.0756 (14)	0.0899 (16)	−0.0045 (9)	−0.0114 (11)	0.0064 (11)
O2	0.0785 (14)	0.0510 (13)	0.1072 (18)	−0.0008 (11)	−0.0232 (12)	−0.0064 (11)
O3	0.0637 (12)	0.0361 (10)	0.0589 (11)	−0.0052 (7)	0.0039 (8)	−0.0025 (7)
O4	0.0527 (11)	0.0494 (12)	0.0677 (12)	0.0033 (8)	−0.0113 (9)	−0.0026 (8)
C1	0.0542 (16)	0.0471 (15)	0.0607 (15)	−0.0005 (11)	0.0027 (12)	0.0022 (12)
C2	0.0519 (15)	0.0492 (15)	0.0498 (14)	−0.0010 (11)	0.0064 (11)	−0.0013 (11)
C3	0.0460 (13)	0.0461 (14)	0.0443 (12)	0.0015 (10)	0.0064 (10)	0.0018 (10)
C4	0.0498 (13)	0.0343 (13)	0.0484 (13)	−0.0022 (9)	0.0097 (11)	0.0010 (9)
C5	0.0473 (14)	0.0442 (14)	0.0497 (13)	0.0013 (10)	0.0064 (11)	0.0017 (10)
C6	0.0563 (16)	0.0438 (15)	0.0592 (15)	0.0077 (10)	0.0061 (12)	−0.0032 (11)
C7	0.0644 (18)	0.0476 (15)	0.0614 (16)	0.0029 (12)	0.0023 (13)	−0.0131 (11)
C8	0.0543 (15)	0.0543 (16)	0.0542 (15)	−0.0005 (12)	0.0029 (11)	−0.0100 (12)
C9	0.093 (2)	0.0542 (17)	0.0689 (19)	−0.0040 (15)	0.0134 (16)	−0.0164 (14)
C10	0.0549 (16)	0.0626 (18)	0.0621 (17)	0.0057 (13)	−0.0009 (13)	0.0123 (13)

### Geometric parameters (Å, °)

**Table:**

N1—O2	1.214 (3)	C4—C5	1.399 (3)
N1—O1	1.225 (3)	C5—C6	1.382 (3)
N1—C1	1.442 (3)	C6—C7	1.391 (4)
O3—C4	1.385 (3)	C6—H6	0.9300
O3—C9	1.431 (3)	C7—C8	1.374 (4)
O4—C5	1.375 (3)	C7—H7	0.9300
O4—C10	1.428 (3)	C8—H8	0.9300
C1—C2	1.314 (3)	C9—H9A	0.9600
C1—H1A	0.9300	C9—H9B	0.9600
C2—C3	1.458 (4)	C9—H9C	0.9600
C2—H2	0.9300	C10—H10A	0.9600
C3—C4	1.395 (3)	C10—H10B	0.9600
C3—C8	1.406 (4)	C10—H10C	0.9600
			
O2—N1—O1	122.5 (2)	C5—C6—H6	120.1
O2—N1—C1	120.7 (2)	C7—C6—H6	120.1
O1—N1—C1	116.7 (2)	C8—C7—C6	120.9 (2)
C4—O3—C9	112.83 (19)	C8—C7—H7	119.5
C5—O4—C10	116.8 (2)	C6—C7—H7	119.5
C2—C1—N1	121.5 (2)	C7—C8—C3	120.3 (2)
C2—C1—H1A	119.2	C7—C8—H8	119.8
N1—C1—H1A	119.2	C3—C8—H8	119.8
C1—C2—C3	125.7 (2)	O3—C9—H9A	109.5
C1—C2—H2	117.1	O3—C9—H9B	109.5
C3—C2—H2	117.1	H9A—C9—H9B	109.5
C4—C3—C8	118.5 (2)	O3—C9—H9C	109.5
C4—C3—C2	119.1 (2)	H9A—C9—H9C	109.5
C8—C3—C2	122.4 (2)	H9B—C9—H9C	109.5
O3—C4—C3	119.2 (2)	O4—C10—H10A	109.5
O3—C4—C5	119.9 (2)	O4—C10—H10B	109.5
C3—C4—C5	120.8 (2)	H10A—C10—H10B	109.5
O4—C5—C6	124.5 (2)	O4—C10—H10C	109.5
O4—C5—C4	115.7 (2)	H10A—C10—H10C	109.5
C6—C5—C4	119.7 (2)	H10B—C10—H10C	109.5
C5—C6—C7	119.7 (2)		
			
O2—N1—C1—C2	−10.7 (4)	C10—O4—C5—C4	179.7 (2)
O1—N1—C1—C2	170.1 (2)	O3—C4—C5—O4	−2.8 (3)
N1—C1—C2—C3	177.7 (2)	C3—C4—C5—O4	−179.41 (19)
C1—C2—C3—C4	168.5 (2)	O3—C4—C5—C6	177.7 (2)
C1—C2—C3—C8	−12.9 (4)	C3—C4—C5—C6	1.0 (4)
C9—O3—C4—C3	−103.4 (3)	O4—C5—C6—C7	−179.6 (2)
C9—O3—C4—C5	79.9 (3)	C4—C5—C6—C7	−0.1 (4)
C8—C3—C4—O3	−177.9 (2)	C5—C6—C7—C8	−0.6 (4)
C2—C3—C4—O3	0.7 (3)	C6—C7—C8—C3	0.4 (4)
C8—C3—C4—C5	−1.3 (3)	C4—C3—C8—C7	0.5 (4)
C2—C3—C4—C5	177.4 (2)	C2—C3—C8—C7	−178.1 (2)
C10—O4—C5—C6	−0.7 (4)		

## References

[bb1] Ballini, R., Bosica, G., Fiorini, D., Palmieri, A. & Petrini, M. (2005). *Chem. Rev.* **105**, 933–971. 10.1021/cr040602r 15755081

[bb2] Bruker (2007). *APEX2* and *SAINT* Bruker AXS Inc., Madison, Wisconsin, USA.

[bb3] Pedireddi, V. R., Sarma, J. A. R. P. & Desiraju, G. R. (1992). *J. Chem. Soc. Perkin Trans. 2*, pp. 311–320.

[bb4] Ranu, B. C. & Banerjee, S. (2005). *Org. Lett.* **7**, 3049–3052. 10.1021/ol051004h 15987202

[bb5] Sheldrick, G. M. (1996). *SADABS* University of Göttingen, Germany.

[bb6] Sheldrick, G. M. (2008). *Acta Cryst.* A**64**, 112–122. 10.1107/S0108767307043930 18156677

[bb7] Wang, C.-D. & Wang, S. (2002). *Synth. Commun.* 32, 3481–3486.

